# Does total lesion prostate‐specific membrane antigen (PSMA) activity on ^68^Ga‐PSMA PET/CT correlate with PSA and prostatectomy histopathological/clinical outcomes in patients with localised primary prostate cancer?

**DOI:** 10.1002/bco2.70015

**Published:** 2025-04-16

**Authors:** Jeremy Cheng, Mohammadmehdi Adhami, Tho Pham, David P. Nadebaum, Ashley Baring, Eldho Paul, Martin Cherk, Jeremy Grummet

**Affiliations:** ^1^ Department of Urology Alfred Health Melbourne Australia; ^2^ Department of Nuclear Medicine and PET Alfred Health Melbourne Australia; ^3^ Alfred Health Melbourne Australia; ^4^ School of Public Health and Preventive Medicine Monash University Melbourne Australia; ^5^ Monash University Melbourne Australia; ^6^ School of Translational Medicine Monash University Melbourne Australia

**Keywords:** clinical outcomes, histopathological outcomes, localised prostate cancer, PSA, PSMA PET/CT, radical prostatectomy, total lesion PSMA

## Abstract

**Objectives:**

To evaluate the relationship between total lesion PSMA (PSMA_TL_), serum PSA, histopathological findings and biochemical recurrence (BCR) in patients with localised prostate cancer (PCa).

**Patients and methods:**

This retrospective study assessed men undergoing ^68^Ga‐PSMA‐11 PET/CT for newly diagnosed or treatment‐naïve PCa localised to the prostate gland. Volumes of interest were manually mapped to derive SUV_max_, SUV_mean_, PSMA‐avid tumour volume and PSMA_TL_. PSMA_TL_ was defined as the product of PSMA‐avid primary tumour volume and SUV_mean_. Spearman correlation tests evaluated associations between PET parameters and PSA, ISUP GG and radical prostatectomy (RP) histopathological outcomes. Associations between PET parameters and clinical outcomes were determined using Cox proportional hazards regression with results presented as HR and 95% CI.

**Results:**

A total of 200 patients were included, with a median age of 68 (IQR 62–73) years, PSA of 9.5 (6.6–13.0) ng/ml and follow‐up of 41 (25–60) months. Median PSMA_TL_ was 29.6 (14.8–54.8) and SUV_max_ 11.0 (6.8–17.9). PSMA_TL_ and SUV_max_ demonstrated a weak correlation with baseline PSA (ρ = 0.334, p < 0.001 and ρ = 0.343, p < 0.001), and PSMA_TL_ showed a weak correlation with PSA density in the RP subgroup (ρ = 0.242, p = 0.021). Among 109 (54.5%) patients undergoing RP, PSMA_TL_ and SUV_max_ showed a weak correlation with ISUP GG (ρ = 0.233, p = 0.015 and ρ = 0.340, p < 0.001). There was a weak correlation between PSMA_TL_ and primary tumour stage (ρ = 0.244, p = 0.010) and lymph node stage (ρ = 0.259, p = 0.007). PSMA_TL_ was significantly higher in those with seminal vesicle involvement (p = 0.011), perineural invasion (p = 0.025) and lymphovascular invasion (p = 0.002). BCR occurred in 46 patients (42%), with a 1% increased risk of BCR per unit increase in PSMA_TL_ (HR 1.01, 95% CI 1.00–1.02, p = 0.011).

**Conclusions:**

PSMA_TL_ correlates with PSA, PSA density, ISUP GG, RP histopathological findings and BCR. As an adjunct to SUV_max_, PSMA_TL_ has the potential to be a useful prognostic tool. Further research is needed to assess its clinical utility.

## 1 INTRODUCTION

Prostate cancer (PCa) is the second most commonly diagnosed cancer in men worldwide.^1^ Serum prostate‐specific antigen (PSA), histopathological International Society of Urological Pathology (ISUP) Grade Group (GG) and clinical tumour stage are imperative in risk stratification of newly diagnosed PCa and thus have significant implications in treatment choice.^2^ Gallium‐68 prostate‐specific membrane antigen (^68^Ga‐PSMA) positron emission tomography/computed tomography (PET/CT) has been shown to provide superior diagnostic accuracy compared to conventional staging CT and bone scan and is now recommended in the staging of intermediate to high‐risk disease, with greater implementation over recent times in this particular cohort.^3^, ^4^, ^5^

PSMA is a cell surface glycoprotein highly expressed in prostate cancer cells.^6^ Maximum standardised uptake value (SUV_max)_ is a parameter conventionally used to quantify the degree of ^68^Ga‐PSMA uptake at sites of prostate cancer on PET/CT. It has been shown that SUV_max_ is directly correlated to the degree of PSMA immunohistochemical expression, and similarly that SUV_max_ increases with serum PSA.^7^, ^8^ Furthermore, SUV_max_ may have utility in differentiating clinically significant PCa from insignificant PCa or benign tissue.^9^, ^10^ Further studies have also demonstrated a correlation between SUV_max_ and Gleason score when staging primary PCa.^11^, ^12^ The utility of SUV_max_ in predicting clinical outcomes has also recently been explored.^13^

A significant limitation of SUV_max_, however, is that it is only a measure of the most intense voxel of activity within a particular region of interest (e.g. the prostate gland) and does not convey any information regarding the total volume of abnormally increased PSMA PET activity.

Total lesion PSMA (PSMA_TL_) is a PSMA PET/CT‐derived measurement quantifying total PSMA uptake in a customizable region of interest such as the whole body or within the prostate gland only. It is analogous to total lesion glycolysis in F‐18 fluorodeoxyglucose (18F‐FDG) PET/CT imaging which conveys a measure of total metabolically active tumour volume.^14^ PSMA_TL_ incorporates PSMA expression and tumour volume into a single PET metric which makes it very attractive for prostate cancer assessment. Despite this, its utility for primary staging remains poorly defined, with prior studies predominantly limited to the metastatic setting in patients receiving Lutetium‐177 PSMA radioligand therapy.^15^, ^16^, ^17^

The primary aim of the present study, therefore, was to evaluate the relationship between ^68^Ga‐PSMA PET/CT PSMA_TL_ and serum PSA levels as well as prostatectomy histopathological and clinical outcomes in patients with PCa localised to the prostate gland only.

## 2 PATIENTS AND METHODS

### 2.1 Study design and participants

This retrospective study analysed ^68^Ga‐PSMA‐11 PET/CT imaging performed at a single, tertiary hospital in Australia between 2015 and 2022. Patients had PET/CT demonstrating disease localised to the prostate gland only and were included if they had either newly diagnosed primary PCa or treatment naïve PCa previously on active surveillance (AS). All patients had transperineal ultrasound‐guided (TP) biopsy‐proven PCa. Patients were excluded if PET/CT was suggestive of locoregional or distant metastases, or if they had received prior treatment for PCa. Data retrospectively recorded and analysed included serial serum PSA levels and PSA subgroups, magnetic resonance imaging (MRI) and PET/CT findings, TP biopsy and radical prostatectomy (RP) pathology findings such as ISUP GG, subsequent treatment and re‐treatment and clinical outcomes such as biochemical recurrence (BCR) and recurrence on repeat PET/CT or conventional staging. The research was conducted in adherence with the Declaration of Helsinki and was approved by the institutional ethics committee (133/16).

### 2.2 Image acquisition

All patients underwent ^68^Ga‐PSMA imaging on a GE Discovery 710 PET/CT scanner (GE Healthcare, Milwaukee WI, United States). Images were acquired from the skull base to the upper thighs following intravenous administration of ^68^Ga‐PSMA‐11. Median injected activity was 163.5 (interquartile range [IQR] 145–182) MBq. The median radiotracer uptake time was 68.5 (60–79) minutes. Low‐dose, non‐contrast‐enhanced CT was performed at 100–140 kVp depending on patient weight, auto‐tube modulation (20–80 mAs) and with a slice thickness of 3.75 mm.

### 2.3 Image analysis

^68^Ga‐PSMA PET/CT images were analysed with MIM software (MIM software Inc, Ohio, United States).

Investigators including two experienced nuclear medicine physicians manually mapped volumes of interest (VOI) that encompassed the prostate gland using the co‐registered CT for anatomical correlation.

Lesions were defined as the region of tumour‐related focal PSMA uptake that was greater than that of surrounding prostate tissue. The VOI was delineated using an SUV threshold of 41% of the prostatic lesion's SUV_max_. This threshold was adopted from a previous study by Draulans et al., who showed the 41 SUV% threshold provided the best approximation of gross tumour volume at histopathology for the ^68^Ga‐PSMA‐11 ligand.^18^

The site of the tumour on TP biopsy was not known to investigators.

PET‐derived measurements calculated were SUV_max_, SUV_mean_, PSMA‐avid tumour volume and PSMA_TL_. PSMA_TL_ was defined as the product of the PSMA‐avid primary tumour volume and SUV_mean_.

### 2.4 Statistical analysis

All analyses were performed using SAS software version 9.4 (SAS Institute, Cary NC, United States). Baseline demographic, histopathological and treatment characteristics of patients were summarised using medians and interquartile ranges or counts and percentages as appropriate. Comparisons of PSMA_TL_/SUV_max_ across PSA subgroups and RP outcomes were performed using Wilcoxon rank‐sum tests for two‐group comparisons and Kruskal‐Wallis tests for comparisons involving more than two groups. Relationships between PET parameters and PSA, PSA density, ISUP GG and certain RP histopathological outcomes were assessed using Spearman rank correlations. Associations between PET parameters and clinical outcomes (time to biochemical recurrence, disease recurrence on imaging, local recurrence and metastatic recurrence) were determined using Cox proportional hazards regression with results presented as hazard ratios (HR) and 95% confidence intervals (CI). All calculated p values were two‐tailed and a p < 0.05 indicated statistical significance.

## 3 RESULTS

Table 1. Baseline patient, histopathological and treatment characteristics (n = 200).

**TABLE 1:** Baseline patient, histopathological and treatment characteristics (n = 200).

	Median (IQR) or n (%)
**Baseline demographics**	
Age at time of PET/CT (years)	68 (62–73)
Follow‐up post PET/CT (months)	41 (25–60)
Deaths*	7 (3.5)
**Baseline PSA at time of PET/CT**	
Serum PSA (ng/ml)	9.5 (6.6–13.0)
PSA density (ng/mL^2^)	0.23 (0.16–0.38)
**Baseline PSA subgroup**	
≤10 (ng/ml)	108 (54)
10–20 (ng/ml)	63 (31.5)
≥20 (ng/ml)	29 (14.5)
**ISUP Grade Group**	
GG 1	3 (1.5)
GG 2	41 (20.5)
GG 3	90 (45)
GG 4	37 (18.5)
GG 5	29 (14.5)
**PSMA parameters**	
SUV_max_	11.0 (6.8–17.9)
SUV_mean_	6.0 (3.8–10.2)
PSMA‐avid metabolic tumour volume (ml)	4.5 (2.0–10.7)
PSMA_TL_	29.6 (14.8–54.8)
**Management**	
Radical prostatectomy	109 (54.5)
• With pelvic lymph node dissection	• 75 (68.8)
• Without pelvic lymph node dissection	• 34 (31.2)
Radiotherapy/brachytherapy	72 (36)
Watchful waiting	9 (4.5)
Active surveillance	5 (2.5)
Androgen deprivation therapy +/− chemotherapy alone	4 (2)
Treatment declined	1 (0.5)
**Radical prostatectomy TNM stage (n = 109)**	
T stage	
pT2	30 (27.5)
pT3	78 (71.6)
pT4	1 (0.9)
N stage	
N0	57 (52.3)
N1	18 (16.5)
NX	34 (31.2)

IQR, interquartile range; PET/CT, positron emission tomography/computed tomography; PSA, prostate‐specific antigen; ISUP, International Society of Urological Pathology; GG, grade group; PSMA, prostate‐specific membrane antigen; SUV_max_, maximum standardised uptake value; SUV_mean_, mean standardised uptake value; PSMA_TL_, total lesion PSMA; TNM, tumour/node/metastasis.

* 7/7 deaths not primarily related to prostate cancer.

Table 2. Correlations between PSMA_TL_, SUV_max_ and PSA, PSA density, ISUP GG, tumour stage and lymph node stage.

**TABLE 2:** Correlations between PSMA_TL_, SUV_max_ and PSA, PSA density, ISUP GG, tumour stage, and lymph node stage.

	PSA	PSA density	ISUP GG (biopsy)	ISUP GG (RP)	Tumour stage	Lymph node stage
All patients (n = 200)						
PSMA_TL_	**0.334** * **, p < 0.001**	0.133, p = 0.096	0.036, p = 0.615	‐	‐	‐
SUV_max_	**0.343, p < 0.001**	**0.303, p < 0.001**	**0.155, p = 0.028**	‐	‐	‐
RP subgroup (n = 109)						
PSMA_TL_	**0.397, p < 0.001**	**0.242, p = 0.021**	0.130, p = 0.179	**0.233, p = 0.015**	**0.244, p = 0.010**	**0.259, p = 0.007**
SUV_max_	**0.325, p = 0.001**	**0.290, p = 0.005**	0.088, p = 0.365	**0.340, p < 0.001**	0.171, p = 0.076	0.173, p = 0.072

PSA, prostate‐specific antigen; ISUP, International Society of Urological Pathology; GG, grade group; RP, radical prostatectomy; PSMA_TL_, total lesion PSMA; SUV_max_, maximum standardised uptake value.

* All results expressed as Spearman ρ correlations.

Table 3. Difference in PSMA_TL_, SUV_max_ between PSA subgroups and prostatectomy outcomes.

**TABLE 3:** Difference in PSMA_TL_, SUV_max_ between PSA subgroups and prostatectomy outcomes.

	PSMA_TL_	p value	SUV_max_	p value
**PSA ≤ 10 vs > 10**		**p = 0.001**		**p < 0.001**
PSA ≤ 10	25.8 (12.2–47.1)		8.8 (6.0–14.5)	
PSA > 10	35.0 (20.0–65.2)		14.0 (8.7–19.5)	
**PSA ≤ 10 vs 10–20 vs ≥ 20**		**p = 0.001**		**p = 0.001**
PSA ≤ 10	25.8 (12.2–47.1)		8.8 (6.0–14.5)	
PSA 10–20	30.1 (17.3–59.0)		11.6 (8.0–18.6)	
PSA ≥ 20	54.1 (29.8–107.8)		18.2 (14.7–23.7)	
**Perineural invasion**		**p = 0.025**		**p = 0.619**
Present	42.8 (16.0–70.6)		11.8 (8.0–19.2)	
Absent	25.8 (14.0–50.2)		11.9 (7.2–19.0)	
**Seminal vesicle involvement**		**p = 0.011**		**p = 0.139**
Present	47.0 (25.7–66.2)		14.5 (8.4–4.6)	
Absent	24.3 (13.2–53.4)		11.0 (7.3–18.6)	
**Lymphovascular invasion**		**p = 0.002**		**p = 0.135**
Present	47.0 (34.5–67.1)		14.2 (9.5–20.8)	
Absent	24.0 (13.0–53.8)		10.9 (7.3–18.6)	
**Extraprostatic extension**		**p = 0.102**		**p = 0.092**
Present	34.5 (16.0–57.4)		12.5 (8.4–19.3)	
Absent	17.6 (10.9–53.8)		9.2 (6.22–18.6)	
**Positive surgical margin**		**p = 0.059**		**p = 0.113**
Present	40.7 (16.8–63.0)		12.8 (9.5–18.9)	
Absent	26.2 (13.5–52.9)		10.5 (7.0–19.1)	
**Complete biochemical response**		**p = 0.149**		**p = 0.055**
Achieved	27.0 (14.9–52.9)		10.5 (7.2–19.3)	
Not achieved	44.8 (14.5–68.5)		14.5 (10.0–18.8)	

PSMA_TL_, total lesion PSMA; SUV_max_, maximum standardised uptake value; PSA, prostate‐specific antigen.

Table 4. Risk of clinical outcome occurrence based on PSMA_TL_ and SUV_max_.

**TABLE 4:** Risk of clinical outcome occurrence based on PSMA_TL_ and SUV_max_.

	BCR		Imaging recurrence		Local recurrence		Metastatic recurrence	
	HR (95% CI)	p value	HR (95% CI)	p value	HR (95% CI)	p value	HR (95% CI)	p value
All patients (n = 200)								
PSMA_TL_	1.0 (1.00–1.01)	0.223	0.99 (0.98–1.01)	0.405	1.0 (0.98–1.01)	0.592	0.99 (0.97–1.01)	0.267
SUV_max_	1.02 (0.99–1.04)	0.129	1.02 (0.99–1.05)	0.201	1.02 (0.98–1.06)	0.327	1.01 (0.96–1.05)	0.827
RP subgroup (n = 109)								
PSMA_TL_	1.01* (1.00–1.02)	**0.011**	1.00 (0.98–1.01)	0.929	1.00 (0.98–1.02)	0.991	1.00 (0.98–1.02)	0.84
SUV_max_	1.01 (0.99–1.04)	0.281	1.02 (0.99–1.05)	0.147	1.02 (0.98–1.06)	0.249	1.01 (0.97–1.06)	0.629

HR, hazard ratio; CI, confidence interval; BCR, biochemical recurrence; imaging recurrence, evidence of disease recurrence on PSMA PET/CT or conventional staging; local recurrence, evidence of disease recurrence in the prostate bed; metastatic recurrence, evidence of disease recurrence outside the prostate bed; PSMA_TL_, total lesion PSMA; SUV_max_, maximum standardised uptake value; RP, radical prostatectomy.

* A HR of 1.01 represents a 1% increase in the risk of BCR per unit increase in PSMA_TL_.

### 3.1 Patient demographics

Baseline patient, histopathological and treatment characteristics are detailed in Table 1. A total of 200 patients underwent ^68^Ga‐PSMA PET/CT, with a median age of 68 (62–73) years and follow‐up of 41 (25–60) months from the time of PET/CT. Just over half of patients had a serum PSA ≤ 10 (54%). ISUP GG 3 was most common (45%) followed by GG 2 (20.5%) and GG 4 (18.5%). Median SUV_max_ was 11.0 (6.8–17.9) and median PSMA_TL_ was 29.6 (14.8–54.8). A total of 109 patients (54.5%) underwent RP, with the majority (68.8%) undergoing pelvic lymph node dissection.

### 3.2 Relationship between PSMA_TL_, SUV_max_ and PSA, PSA density, biopsy ISUP GG

As illustrated in Table 2, both PSMA_TL_ and SUV_max_ demonstrated a weak correlation with baseline PSA (Spearman ρ = 0.334, p < 0.001 and ρ = 0.343, p < 0.001, respectively). This was also the case in the RP subgroup (ρ = 0.397, p < 0.001 and ρ = 0.325, p = 0.001, respectively). Similarly, there was a weak correlation between PSMA_TL_ and PSA density in the RP subgroup (ρ = 0.242, p = 0.021), but not in the overall cohort. There was a statistically significant but weak correlation between SUV_max_ and biopsy ISUP GG (ρ = 0.155, p = 0.028), but not with PSMA_TL_. Whilst these correlations reached statistical significance, they are generally considered ‘weak’ associations; therefore, the correlation coefficient alone does not necessarily translate to a meaningful clinical implication. There was a significant difference in both PSMA_TL_ and SUV_max_ across PSA subgroups ≤10 vs > 10 and ≤10 vs 10–20 vs ≥ 20, as detailed in Table 3.

### 3.3 Relationship between PSMA_TL_, SUV_max_ and prostatectomy histopathological outcomes

In the RP subgroup, both PSMA_TL_ and SUV_max_ demonstrated a weak correlation with RP specimen ISUP GG (ρ = 0.233, p = 0.015 and ρ = 0.340, p < 0.001, respectively). There was also a weak correlation between PSMA_TL_ and both the primary tumour stage (ρ = 0.244, p = 0.010) and lymph node stage (ρ = 0.259, p = 0.007).

As detailed in Table 3, a significant difference in median PSMA_TL_ was observed between the presence or absence of seminal vesicle involvement (SVI) (p = 0.011), perineural invasion (PNI) (p = 0.025) and lymphovascular invasion (LVI) (p = 0.002), but not extraprostatic extension (EPE) (p = 0.102) or positive surgical margins (PSM) (p = 0.059).

There was no association between SUV_max_ and any other histopathological outcomes aside from ISUP GG.

### 3.4 Relationship between PSMA_TL_, SUV_max_ and clinical outcomes

After a median follow‐up of 40 (20–62) months post‐RP, BCR was observed in a total of 46 patients (42%). There was a 1% increased risk of BCR per unit increase in PSMA_TL_ (HR 1.01, 95% CI 1.00–1.02, p = 0.011). Furthermore, there was a significant difference in PSMA_TL_ between patients who had BCR compared to those who did not (44.9 ± 35.1 vs 36.4 ± 30.1, respectively, p = 0.046).

Aside from this, there were no other significant associations between PSMA_TL_ or SUV_max_ and BCR, disease recurrence on imaging, local disease recurrence or metastatic recurrence in either the overall cohort or RP subgroup (Table 4).

There was also no significant difference in PSMA_TL_ or SUV_max_ in patients who did or did not have complete biochemical response post‐RP (Table 3).

## 4 DISCUSSION

Given that PSMA_TL_ is a composite function of both SUV_mean_ and total PSMA‐avid tumour volume, we expected PSMA_TL_ may have similar utility compared to SUV_max_ alone. Our retrospective study demonstrated that PSMA_TL_ is a promising PET/CT parameter with potential for important clinical applications. Similar to SUV_max_, PSMA_TL_ appears to correlate with serum PSA and PSA density and may be used to help predict PSA subgroup and thus assist in risk stratification of localised PCa. Furthermore, we demonstrated a significant relationship between PSMA_TL_ and important RP histopathological features such as SVI, PNI and LVI, that may have a substantial impact on clinical outcomes.^19^ This is ultimately reflected by the fact that PSMA_TL_ may play a role in predicting BCR following RP.

SUV_max_ has previously been shown to correlate with serum PSA.^8^, ^11^, ^12^ In a retrospective analysis of 201 patients with newly diagnosed PCa, Onal et al. demonstrated a ‘moderate’ correlation between SUV_max_ and PSA.^11^ Uprimny et al. reported a significantly higher SUV_max_ in 90 patients with PSA ≥ 10.0 ng/ml compared to <10 ng/ml.^12^ This was in a similar population to ours, with patients undergoing primary staging ^68^Ga‐PSMA PET/CT and a comparable median serum PSA of 9.7 ng/ml (compared to 9.5 ng/ml in our study). Our study demonstrated similar relationships between PSA and SUV_max_ and more notably PSA and PSMA_TL_. Compared to our analysis, both these studies included patients with locoregional disease on PET/CT (with Uprimny et al. also including metastatic disease), and the median PSA was significantly higher in the study performed by Onal et al (20.3 ng/ml). Similar volumetric PET parameters have been explored with other PET‐derived biomarkers such as total metabolic tumour volume (TMTV) in other PET tracers. Fragkiadaki et al. demonstrated positive correlations between TMTV and SUV_max_ with PSA levels in their study of 104 patients with BCR who underwent both ^18^F‐PSMA‐1007 and ^18^F‐choline PET/CT.^20^ Despite these differences, our results suggest that similar to SUV_max_, PSMA_TL_ may be used to predict PSA levels and subgroups in patients with localised PCa, and similar to above, potentially may be extrapolated to those with locoregional and metastatic disease, as well as in patients with BCR.

PSMA_TL_ may correlate with or help predict important RP histopathological outcomes following prostatectomy. We demonstrated a ‘weak’ correlation between both PSMA_TL_ and SUV_max_ with prostatectomy ISUP GG. Although this reached statistical significance, the clinical relevance of a ‘weak’ association needs further evaluation, as statistical significance does not necessarily translate to a clinically significant result. Future studies are required to further explore the strength of this correlation, and therefore, also its potential clinical utility. Similarly, Demirci et al. reported a ‘strong’ correlation between SUV_max_ and ISUP GG in their study of 141 patients with localised disease, with a Pearson ρ coefficient of 0.66.^9^ This difference in magnitude of correlation may be partially accounted for by the fact that our data were heavily skewed towards ISUP GG 2 and 3 disease (24% and 49%, respectively), whilst patients in the above study were relatively more evenly spread across all Grade Groups.

Wang et al. explored the role of PSMA_TL_ in predicting RP outcomes in a retrospective analysis of 186 patients with D'Amico intermediate to high‐risk primary PCa.^21^ They reported a significant association between positive surgical margins and PSMA_TL_ (odds ratio [OR] 1.007, p = 0.021) as well as with SUV_max_ (OR 1.026, p = 0.039). There was, however, no significant association between PSMA_TL_ and ISUP GG, and they did not explore other histopathological features such as PNI, SVI, LVI or EPE. To our knowledge, this is the only other study exploring the role of PSMA_TL_ in predicting RP histopathological outcomes and reflects the promising findings from our analysis. Similar studies have otherwise only explored SUV_max_, with Roberts et al. reporting a significant association with Gleason Score, T‐stage and positive surgical margins.^22^ Due to the composite nature of PSMA_TL_, we expect that future studies would demonstrate a similar, if not more substantial, relationship with histopathological outcomes when compared to SUV_max_ alone. In a prospective trial by Li et al. comparing 18F‐DCFPyL PSMA PET with limited MRI to multiparametric MRI alone, PSMA PET showed higher specificity in detecting EPE and higher sensitivity in detecting lymph node involvement.^23^ This suggests the potential to further improve the staging accuracy of PSMA PET by combining radiomic measures such as PSMA_TL_ with the anatomical detail provided by MRI.

To the best of our knowledge, no previous study has shown PSMA_TL_ to be a predictor of clinical outcomes post‐RP. Wang et al. explored the role of PSMA_TL_ in predicting BCR‐free survival after a median follow‐up of 38 months post‐RP, but found no significant relationship.^21^ Instead, SUV_max_ alone was a significant predictor of BCR‐free survival (HR 1.015, 95% CI 1.004–1.026, p = 0.008). Similarly, de Bie et al. reported SUV_max_ as a predictor for BCR in 238 patients post‐robot‐assisted RP, with SUV_max_ > 10 a significant cut‐off value.^13^ Roberts et al. also found SUV_max_ to be a predictor of progression‐free survival.^22^ As a prognostic tool, PSMA_TL_ has otherwise only been explored in patients with metastatic disease receiving Lutetium radioligand therapy and has been shown to be a predictor for overall survival.^16^, ^17^ Our study is the first to demonstrate the potential use of PSMA_TL_ in localised PCa as a predictor of clinical outcomes post‐RP, in the form of BCR. Although we did not show a significant association with other outcome measures (such as disease recurrence, local or metastatic recurrence), it is worth noting that the aforementioned studies similarly demonstrated a significant relationship between SUV_max_ and only one clinical outcome each. This suggests that whilst the evidence of both PSMA_TL_ and SUV_max_ in predicting clinical outcomes post‐BCR is evolving, perhaps one outcome could be used as a surrogate for another whilst data continues to mature.

A major limitation of this study is the retrospective nature of the analysis. This, coupled with being a single‐centred study, could potentially have led to bias. However, findings are consistent with that already published in the literature. Despite having an overall sample size comparable to the existing literature, there was also a relative predominance towards intermediate‐risk factors, with the majority of patients having PSA < 10 ng/ml (54%) and ISUP GG ≤ 3 disease (67%). As a result, this could limit the generalisability of our findings in other patient cohorts. Prospective studies, at other institutions, with different patient populations, are required to assess the applicability and utility of PSMA_TL_ in other patient cohorts. Furthermore, we excluded patients with locoregional disease on ^68^Ga‐PSMA PET/CT. This could potentially have led to sampling bias, particularly in patients with N1 disease on lymph node dissection despite no evidence on PET/CT.

A major strength of this study is the long follow‐up time. The median post‐RP follow‐up of 40 (20–62) months was longer than all the aforementioned studies exploring clinical outcomes (19.5, 32 and 38 months).^13^, ^21^, ^22^ Furthermore, patients were closely followed up, with a median of seven (5–11) post‐RP serum PSA levels performed. As a result, a considerable number of BCR incidents and imaging recurrences were detected, strengthening the analysis of PSMA_TL_ as a predictor of clinical outcomes.

## 5 CONCLUSIONS

In conclusion, we demonstrate that ^68^Ga‐PSMA‐11 PET/CT PSMA_TL_ is a promising parameter that, similar to SUV_max_, may correlate with PSA levels, PSA density and ISUP GG. PSMA_TL_ also showed an association with both prostatectomy histopathological findings and clinical outcomes. Utilised as an adjunct to SUV_max_, PSMA_TL_ has the potential to be a useful prognostic tool. These initial results from a single‐centred study should be further explored in prospective studies to assess the potential application of PSMA_TL_ in routine clinical practice.

## AUTHOR CONTRIBUTIONS

Conceptualisation and Study Design: TP, DN, MC, JG; Image Analysis: TP, DN, MC; Data acquisition: JC, AB; Statistical Analysis and Interpretation: JC, MA, EP; Manuscript Writing: JC, MA; Manuscript Review: JC, MA, TP, DN, AB, EP, MC, JG.

All authors have reviewed the manuscript and approved the final version for publication.

## CONFLICT OF INTEREST STATEMENT

The authors have no relevant financial or non‐financial interest to disclose.
